# Broken Instruments in Root Canals

**Published:** 1891-03

**Authors:** Wm. Conrad

**Affiliations:** St. Louis.


ARTICLE IV.

### BROKEN INSTRUMENTS IN ROOT CANALS.

BY WM. CONRAD, D. D. S., ST. LOUIS.

Read before the St. Louis Dental Society, St. Louis, March 3d, 1891.

How to remove them, is a question often asked at dental meetings with never a satisfactory answer to the query. Judging from my own experience, there is no set rule by which we can act; circumstances must suggest the method we are to follow in these difficult cases. We are never certain as to the degree of success we will have, and for this reason it should be our endeavor to avoid everything that will leave the case in a worse condition than it was when we commenced to work.

When this subject was first given to me I had hoped to be able to present something of practical value, but the more I tried to solve the problem, the more difficult became the subject, and as a result, I have only a few general ideas to offer for your consideration to-night. It will be rather to tell you to avoid these accidents, than to enlighten you on the point as to how to remove them when you have the instrument in the canal.

In operating upon pulpless teeth, the best thing to do in order to insure your future comfort, is not to break the instrument off; but this would be out of all human power, as such things have happened and will happen again to the most careful of dentists, although at the time of such accidents the feeling is that carelessness has been the cause of our misfortunes.

A dentist, who says he never breaks instruments in root canals, must be one who never treats and fills pulpless teeth. I believe there are some such people still alive, although their numbers are not so numerous as formerly. A boast of this kind can never raise the reputation of the operator in the estimation of the large class of progressive dentists who are every day trying to do their best for those who trust their ability to save the natural teeth.

I must be in the position of one who has not the requisite amount of skill to properly handle instruments, as I have broken quite a number of them in roots during my years of practice.

The kind of broaches to use in order to get the best results, is an important consideration, and not as easily answered as might at first be imagined. The individual preferences of the different dentists must be the guide to a proper selection.

For general use, I prefer the Jeweler's Swiss broach, Nos. 6-8, temper drawn to a spring, at the time of using, dressing the surface with an emery disc and burnishing with pliers. This gives a smooth, flexible instrument now ready for use. Sometimes I have to sharpen the point for difficult work.

Gold, platinum, gold and platinum, etc., have been recommended as materials for broaches, but I cannot see any advantage in their use over the steel instruments. They will break as readily as steel, and as it is not from the corroding of the piece left in the canal that we have the most to fear, we can gain nothing by their use. It is from a blocking up of the canal in such a way that the root cannot be properly treated and filled, or by forcing the point through the apical foramen, that we expeet the greatest danger.

How to use them is a subject for our constant study, and something we all think we know until an accident happens, then we begin to invent excuses for our lack of knowledge. Never use a broach many times. If a new one is selected for each case it might save us hours of annoyance, and perhaps some professional credit. Of course we use a new one every time the case seems to demand it, but to our sorrow this is not enough. Give up the old one, no matter how well it may be adapted for the work on hand, before we are compelled to make a change from necessity. Care in this one particular will save us trouble.

Never twist a broach in a canal; although we may be sorely tempted so to do, we are likely to twist once too often. There are more barbed broaches left in roots than all other instruments combined. I am glad to learn that their use is going out of style, and I hope they may never become a necessity again.

From my observation, the greatest number of dentists make use of mechanical means to get the pieces out of the canals. This method is all very well, if we succeed, but in case we do not accomplish our object, it leaves the case in a much worse condition than if we had never made the attempt. If the effort at removal has not been successful the broken instrument has been forced more firmly into the canal, leaving a space beyond which cannot be treated or filled, setting up a low form of inflammation, and being always a constant reminder that abscess is liable to follow, unless the end of the root becomes encysted, and to my mind a permanent case of this kind of relief is rare, and not to be relied on for safety. If the instrument is forced through the apical foramen, this condition to me would mean the final loss of the tooth. In such cases as this, gold; platinum, gold and platinum broaches do not make a good root filling, as some of our brothers a few years ago stated as a reason for the use of other metals than steel in the manufacture of broaches.

The action of chemicals upon metals seems to me to be the ideal plan for solving this difficulty. Some acid which will dissolve the broken instrument, thus doing away with the necessity of disturbing it by any mechanical effort, and when it has been sufficiently softened, allowing us to wash out the remaining debris, would be, to my way of thinking, a blessing to dentists of to-day. How to do this is a question I would like to have answered. Such a chemical should act more rapidly upon the instrument than upon the tooth substance, and there will be the difficulty, as everything I have used requires a greater quantity in contact with the metal than we get there, or will act too rapidly upon the root.

Chemicals used for this purpose are numerous. None of them can be relied on at all times. Common salt has been recommended, but it will not do the work. Hydrogen di oxide pumped into the canal and around the piece, should do it, but will not. Bichloride of mercury, forced around the broach, does not seem to care for the task.

Sulphuric acid I have tried with fairly good results. It certainly will do no harm, and is worth a trial in case of necessity, as it softens calcified pulp matter and gives you more room to better treat the roots.

Nitric acid is one of the most certain means we have to use, although the danger in its use makes care a great necessity. I believe I can remove any steel instrument from a root by its use. I use it diluted, and the gold broach wrapped with cotton as my means of applying it. In some desperate cases I have taken advantage of it, and have not been sorry.

In conclusion, let me say, it is a misfortune that sometimes our patients will go visiting the other dentist, just when we would prefer all of their company ourselves, and it is a consolation for some of us to know that it is not always the most unskillful dentist who pushes the broach the furthermost through the apical foramen.

I certainly would not tell the patient the instrument was in the root; it will do no good, and many times may do harm, both to patient and dentist.

My advice would be to "*Keep it dark and do your best.*" We always lose money on such cases where the "other fellow" did not break the instrument, as it is hardly worth $12.00 per hour to the patient, our efforts to save our own reputation.—*Archives of Dentistry.*

